# Immunometabolic profiling in menopausal women with multiple sclerosis: the role of adipokines and hormone therapy

**DOI:** 10.1136/bmjno-2025-001295

**Published:** 2025-11-30

**Authors:** Laura Juutinen, Katja Ahinko, Sanna Hagman, Tarja Kokkola, Sari Kärkkäinen, Olli Jääskeläinen, Mari Hämäläinen, Pabitra Basnyat, Julia Vistbacka, Sanna-Kaisa Herukka, Eeva Moilanen, Marja-Liisa Sumelahti

**Affiliations:** 1 Faculty of Medicine and Health Technology, Tampere Universities, Tampere, Finland; 2 Department of Sensory, Neural, and Musculoskeletal Medicine, Tampere University Hospital, Tampere, Finland; 3 Department of Obstetrics and Gynecology, Tampere University Hospital, Tampere, Finland; 4 Neuroimmunology Research Group, Faculty of Medicine and Health Technology, Tampere Universities, Tampere, Finland; 5 Neurology, University of Eastern Finland Institute of Clinical Medicine, Kuopio, Finland; 6 The Immunopharmacology Research Group, Faculty of Medicine and Health Technology, Tampere Universities, Tampere, Finland; 7 Department of Neurology, Neurocenter, Kuopio University Hospital, Kuopio, Finland; 8 Tampere University Hospital, Tampere, Finland

**Keywords:** MULTIPLE SCLEROSIS, MENOPAUSE, HORMONE REPLACEMENT THERAPY, ADIPOKINES, INFLAMMATION

## Abstract

**Background:**

Menopausal changes in adipose tissue distribution and adipokine profiles may influence immune activity in multiple sclerosis (MS). We investigated relationships between adipokines, inflammation and disease severity in menopausal women with MS and evaluated changes in adipokines during menopausal hormone therapy (MHT).

**Methods:**

16 menopausal women with MS (participants with MS, PwMS) and 15 age-matched healthy controls were assessed for the associations of adipokines with inflammatory markers, and clinical, radiological and fluid biomarkers of MS severity. Adipokine levels were monitored over 1 year of oral MHT in baseline-controlled design.

**Results:**

In PwMS, body mass index and leptin-to-adiponectin ratio correlated with circulating high-sensitivity C reactive protein (hs-CRP), tumour necrosis factor (TNF)-α and interleukin-6 (ρ=0.51–0.66, p<0.05). The associations with hs-CRP and TNF-α were independent of age, disease duration, follicle-stimulating hormone and vitamin D. Serum vitamin D inversely correlated with hs-CRP, TNF-α and interferon-γ (ρ=–0.64–0.65, p<0.01). Adipsin showed strong correlation with neurofilament light chain (ρ=0.72, p=0.002) and decreased during MHT (3 months: p=0.007; 12 months: p=0.04).

**Conclusions:**

Adipokine imbalance and lower vitamin D levels were associated with systemic inflammation in PwMS. Adipsin emerged as a promising biomarker, with potential relevance for disease monitoring and therapeutic modulation via hormonal pathways. These findings support further exploration of multibiomarker profiling to guide personalised care in menopausal MS.

**WHAT IS ALREADY KNOWN ON THIS TOPIC**

- The menopausal transition is associated with proinflammatory metabolic changes, some of which can be partially reversed with menopausal hormone therapy. However, the implications of this in multiple sclerosis (MS) remain largely unexplored.

**WHAT THIS STUDY ADDS**

- In menopausal women with MS, obesity, imbalanced adipokine secretion and lower vitamin D were linked to increased systemic inflammation. Complement-related adipokine, adipsin, emerged as a potential biomarker of MS severity and was reduced during menopausal hormone therapy.

**HOW THIS STUDY MIGHT AFFECT RESEARCH, PRACTICE OR POLICY**

- The metabolic-inflammatory axis might contribute to disease progression in menopausal women with MS by sustaining chronic peripheral immune activation. The potential hormonal modulation of the complement system warrants further investigation in the future.

## Introduction

 Hormonal transitions across the female lifespan, such as puberty and pregnancy, are associated with changes in multiple sclerosis (MS) activity.^1^ Emerging evidence suggests that natural menopause may coincide with a transition to a more progressive phase of MS. This is indicated by reduced relapse rates and MRI activity, as well as lower grey matter volumes and increased serum neurofilament light chain (NfL) levels observed after menopause.^2^,^5^

Menopausal transition leads to increased visceral fat accumulation, adipose tissue dysfunction and altered adipokine secretion.^6^,^8^ These changes promote chronic systemic inflammation and may increase the risk of subsequent neurodegeneration, a concern that is particularly relevant in MS given that immune dysregulation and neuroinflammation drive disease progression.^9^,^11^ Yet, the immunometabolic role of adipose tissue in menopausal women with MS remains largely unexplored.

Adipocytes secrete hormone-like molecules adipokines, such as leptin, adiponectin, resistin and adipsin, that play a vital role in energy metabolism and are also increasingly recognised as modulators of immune responses in both innate and adaptive immunity.^12^ In addition, adipokines can regulate neuroinflammation, glial cell activation and oxidative stress—key processes in MS progression.^13 14^ Proinflammatory adipokine leptin has been associated with disease activity and disability progression in MS, whereas the role of anti-inflammatory adiponectin is less clear.^10 15^ Notably, adipsin, a component of the alternative complement pathway, has emerged as a potential biomarker of neurodegeneration and disease activity in MS.^16^

Menopausal hormone therapy (MHT) can mitigate menopause-associated abdominal fat accumulation,^8^ but its effects on adipokines remain inconsistent. While some studies report no significant changes in leptin or adiponectin levels with MHT, others suggest that the impact may vary depending on the hormone type and the route of administration.^17^ Whether MHT can modulate adipokine secretion in menopausal women with MS remains an open question.

This prospective observational case–control study investigated how menopause-associated metabolic changes—particularly increased adiposity and altered adipokine secretion—modulate systemic inflammation in women with MS and healthy controls (HCs), and how these factors related to MS severity. Changes in adipokine profiles over 1 year of oral MHT were assessed using a baseline-controlled design.

## Methods

### Study population and procedures

**Figure 1:**
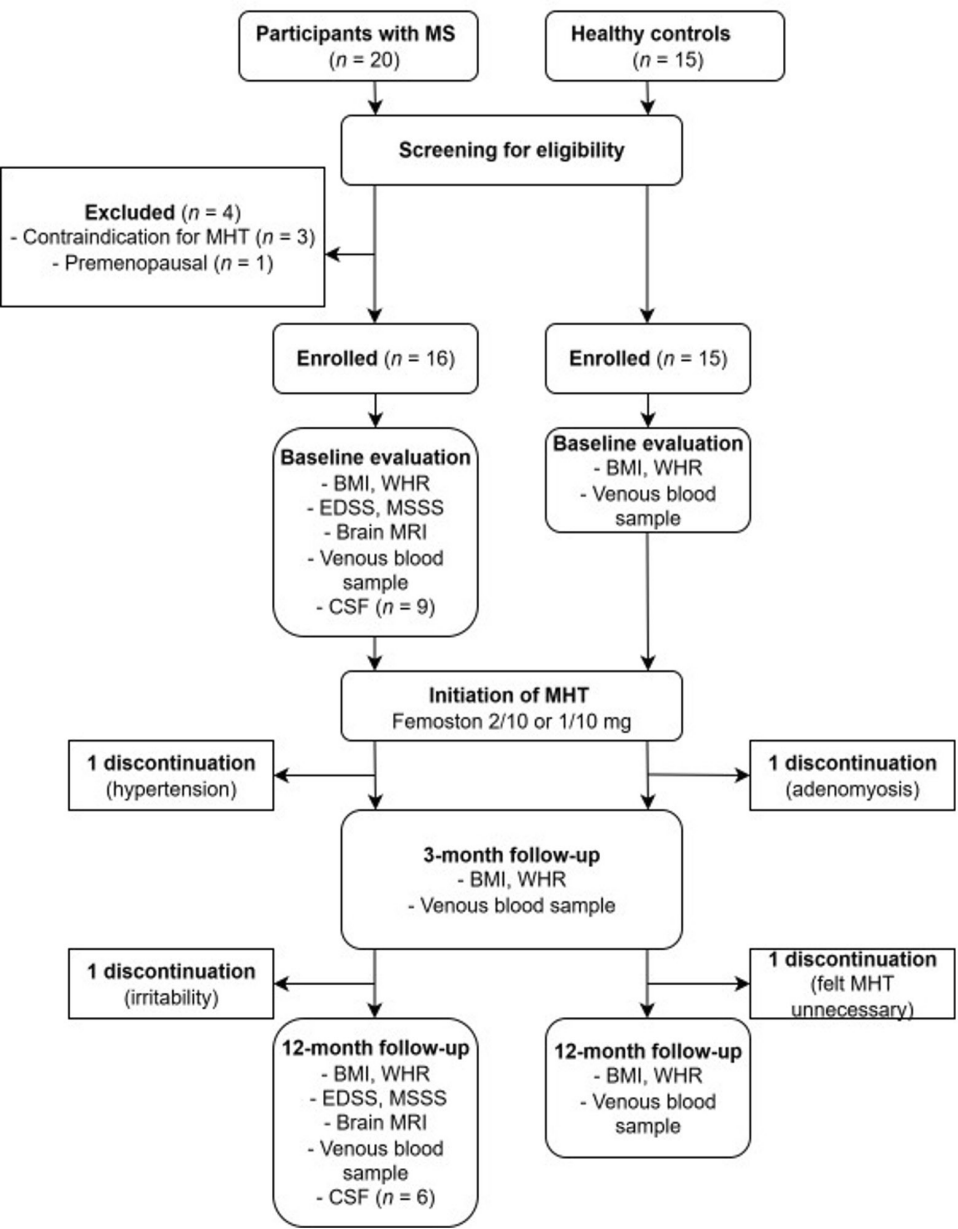
Flow chart of study cohort and procedures. BMI, body mass index; CSF, cerebrospinal fluid; EDSS, Expanded Disability Status Scale; MHT, menopausal hormone therapy; MS, multiple sclerosis; MSSS, Multiple Sclerosis Severity Score; WHR, waist-hip ratio.

This 1-year follow-up study included 35 perimenopausal and postmenopausal women: 20 participants with MS (PwMS) and 15 age-matched HCs (figure 1). PwMS were recruited between August 2015 and September 2017 from the Neurology Outpatient Clinic at Tampere University Hospital and local MS organisations. HCs were recruited from staff at Tampere University and Tampere University Hospital. This study was reported in accordance with the Strengthening the Reporting of Observational Studies in Epidemiology guidelines.^18^

Baseline menopausal status was determined by a gynaecologist based on clinical examination, transvaginal ultrasound and serum levels of oestradiol, follicle-stimulating hormone (FSH) and luteinising hormone (LH). Participants with FSH levels <30 IU/L were classified as perimenopausal, whereas those with higher levels were considered postmenopausal. Body mass index (BMI) was calculated as weight (kg) divided by height squared (m²), and waist-to-hip ratio (WHR) as waist circumference divided by hip circumference to assess fat distribution. MS severity was evaluated using the Expanded Disability Status Scale (EDSS) and the Multiple Sclerosis Severity Score (MSSS),^19^ assessed by the same neurologist. HCs had no history or clinical signs of neurological disease.

The inclusion criteria for PwMS were a confirmed diagnosis of relapsing-remitting MS (RRMS) or secondary progressive MS (SPMS) and mild to moderate disability (EDSS <6). Participants were allowed to continue first-line disease-modifying therapies (DMTs), including interferon-β preparations, glatiramer acetate, teriflunomide and dimethyl fumarate. To reduce population heterogeneity, individuals receiving second-line treatments (fingolimod, natalizumab or alemtuzumab at the time of data collection) were excluded.

Following screening, four PwMS were excluded (figure 1). The final study population included 16 PwMS and 15 HCs, all of whom initiated oral MHT consisting of 1 or 2 mg oestradiol hemihydrate combined with cyclic 10 mg dydrogesterone (Femoston). This formulation has demonstrated beneficial effects on metabolic parameters and fat distribution.^20^ The oestradiol dose was selected by a gynaecologist based on menopausal status and symptom severity. Treatment efficacy and tolerability were assessed at 3 months, with dose adjustments made as needed.

### Laboratory measurements

Venous blood samples were collected at baseline, 3 months and 12 months. With separate consent, lumbar punctures were performed in nine PwMS at baseline and repeated in six of them at 12 months.

Serum levels of oestradiol, FSH and LH for menopausal staging were measured at baseline using electrochemiluminescence immunoassays (Roche Diagnostics, Mannheim, Germany). Serum 25-hydroxyvitamin D (hereafter vitamin D) levels were determined as an indicator of overall vitamin D status using a direct competitive chemiluminescence immunoassay (Liaison 25 OH Vitamin D Total Assay, DiaSorin, Stillwater, Minnesota, USA) on the Liaison XL immunoanalyser.

Four adipokines (leptin, resistin, adiponectin and adipsin), nine inflammatory markers and three MS-related biomarkers were analysed in all peripheral blood and cerebrospinal fluid (CSF) samples collected at baseline and follow-up time points. Inflammatory markers included high-sensitivity C reactive protein (hs-CRP), tumour necrosis factor-α (TNF-α), interferon-γ (IFN-γ), C-C motif chemokine ligand 2 (CCL2), C-X-C motif chemokine ligand 8 (CXCL8), interleukin (IL)-1β, IL-6 and IL-10. MS-related biomarkers included NfL, glial fibrillar acidic protein (GFAP) and chitinase 3-like 1 (CHI3L1).^21^

ELISA was used to quantify plasma and CSF levels of adipokines, hs-CRP, CCL2 and CHI3L1 (R&D Systems Europe, Abingdon, UK) and CXCL8 (BD Biosciences, Erembodegem, Belgium). Single-molecule array (SIMOA) technology was used to measure serum and CSF levels of TNF-α, IFN-γ, IL-1β, IL-6 and IL-10 (SIMOA HD-X, Quanterix Corporation, Billerica, Massachusetts, USA), as well as NfL and GFAP (SIMOA HD-1). Samples were centrifuged at 10 000×g for 5 min at 22°C, and the concentrations were quantified using commercial kits from Quanterix.

BMI-normalised plasma adipokine concentrations were also calculated, although absolute values were used in statistical analyses unless otherwise specified. The leptin-to-adiponectin ratio was calculated to reflect adipose tissue dysfunction.^22^

### MRI

Brain MRI was performed on PwMS at baseline and 12 months using the same 1.5 Tesla scanner (GE Healthcare Signa HDxt). The imaging protocol included T1-weighted and T2-weighted sequences, fluid-attenuated inversion recovery (FLAIR), diffusion-weighted imaging and gadolinium-enhanced T1-weighted sequences. A neuroradiologist blinded to the clinical data analysed all scans. FLAIR images (voxel size 1×1×1 mm) were used to quantify whole brain volume (WBV) and white matter lesion volume (LV) using MSmetrix software (icometrix, Leuven, Belgium).^23^ Volumetric analysis failed for two baseline and three 12-month scans because of technical issues.

### Statistical analysis

Continuous variables were summarised as mean (SD) or median (IQR), and categorical variables as frequency (percentage). Between-group comparisons at baseline were performed using the Mann-Whitney U test for continuous variables and Fisher’s exact test for categorical variables. Spearman’s rank correlation was used to assess associations between variables.

Multiple linear regression analyses were conducted to evaluate the associations of BMI and the leptin-to-adiponectin ratio with inflammatory markers, adjusting for age, disease duration since the confirmed diagnosis of MS, FSH or vitamin D (one covariate at a time owing to limited sample size). The residuals were checked for approximate normality.

Given the preliminary and exploratory nature of the study and a relatively small sample size, corrections for multiple comparisons were not applied to avoid the loss of statistical power and increased risk of type II errors.

Within-group changes in adipokine levels over time were assessed using the Wilcoxon signed-rank test. Only complete paired data were included in the longitudinal analyses. Non-parametric and exact tests were used due to the small sample size and skewed data distributions. All analyses were performed using SPSS Statistics V.28.0 (IBM), with a significance threshold of p<0.05.

## Results

### Baseline characteristics

The baseline characteristics of the study participants are summarised in table 1. PwMS were more often classified as postmenopausal (FSH>30 IU/L; p=0.07). The majority of participants in both groups were overweight or obese (BMI ≥25.0 kg/m²) and had abdominal obesity (WHR ≥0.85). None of the PwMS and 27% of HCs had arterial hypertension. No participants had type 2 diabetes or were using statins. Vitamin D supplementation was significantly more common among PwMS compared with HCs (88% vs 47%, p=0.02), with a higher average daily dose (77 µg vs 13 µg, p<0.001).

**Table 1:** Baseline characteristics of the participants with MS and healthy controls

Characteristic	Participants with MS (n=16)	Healthy controls (n=15)	P value*
Age (years)	51.4±2.7 (45−52)	50.6±3.9 (45−58)	0.44
Menopausal status†			0.07
Perimenopausal	3 (19%)	8 (53%)	
Postmenopausal	13 (81%)	7 (47%)	
E2 (nmol/L)	0.04 (0.005–0.2)	0.1 (0.0–0.8)	0.19
FSH (IU/L)	67.2 (44.9–79.3)	21.8 (6.9–74.3)	0.11
LH (IU/L)	40.0 (31.6–46.0)	19.2 (6.6–36.7)	0.07
BMI (kg/m²)	27.7±5.9 (18.0−41.2)	27.0±2.9 (22.8−33.5)	0.95
Overweight (BMI 25.0–29.9 kg/m²)	4 (25.0%)	10 (66.7%)	0.02
Obese (BMI ≥30.0 kg/m²)	5 (31.3%)	2 (13.3%)	0.22
Overweight or obese (BMI ≥25.0 kg/m²)	9 (56%)	12 (80%)	0.15
WHR	0.86±0.05 (0.77−0.94)	0.86±0.04 (0.76−0.91)	0.89
Abdominal obesity (WHR ≥0.85)	9 (60%)	11 (73%)	0.12
Vitamin D supplement use	14 (88%)	7 (47%)	0.02
Daily dose (µg)	77±42.3 (0–150)	13±25.6 (0–100)	<0.001
Current smokers	2 (13%)	1 (7%)	1.0
Pregnancies	1.6±1.7 (0−6)	2.1±1.2 (0−4)	0.19
MS type			
RRMS	12 (75%)		
SPMS	4 (25%)		
Disease duration (years)	15.2±9.0 (3−34)		
EDSS	2.75 (2.5–4.5)		
Disease-modifying therapy, n			
Interferon -β	3 (19%)		
Glatiramer acetate	2 (12.5%)		
Dimethyl fumarate	2 (12.5%)		
None	9 (56%)		

Continuous variables are expressed as mean±SD (range) except for hormonal values and EDSS as median (IQR). Categorical variables are expressed as frequency (%).

* Mann-Whitney U test was used for continuous variables and Fisher’s exact test for categorical variables. Level of significance p<0.05.

† Participants with FSH higher than 30 IU/L were classified as postmenopausal.

BMI, body mass index; E2, oestradiol; EDSS, Expanded Disability Status Scale; FSH, follicle-stimulating hormone; LH, luteinising hormone; MS, multiple sclerosis; RRMS, relapsing remitting MS; SPMS, secondary progressive MS; WHR, waist-to-hip ratio.

All PwMS had been relapse-free for at least 6 months prior to enrolment, and only one participant exhibited a gadolinium-enhancing lesion on baseline MRI. DMTs were initiated between 5 months and 7 years before baseline and were continued throughout the follow-up period.

### Baseline differences in adipokine levels between PwMS and HCs

There were no statistically significant differences in plasma adipokine levels between PwMS and HCs (table 2, online supplemental figure 1). Normalising adipokine concentrations by BMI did not alter the results (online supplemental table 1). Serum vitamin D levels were significantly higher in PwMS compared with HCs (p=0.004; table 2, online supplemental figure 1), likely due to more intensive supplementation as indicated in table 1.

**Table 2:** Circulating levels of adipokines and vitamin D at baseline presented as median (IQR)

	Participants with MS (n=16)	Healthy controls (n=15)	P value*
Leptin (ng/mL)	40.0 (17.6–80.6)	24.1 (20.7–58.7)	0.65
Adiponectin (µg/mL)	5.99 (4.42–7.32)	4.64 (3.43–6.95)	0.18
Leptin-to-adiponectin ratio	5.70 (3.03–13.21)	5.17 (3.64–14.60)	0.80
Resistin (ng/mL)	12.3 (10.9–14.1)	12.3 (10.3–15.8)	0.88
Adipsin (µg/mL)	0.71 (0.61–0.93)	0.86 (0.73–1.03)	0.20
Vitamin D (nmol/l)	92.6 (80.8–127.5)	73.0 (57.5–84.2)	**0.004**

Level of significance p<0.05.

* Mann-Whitney U test.

MS, multiple sclerosis.

### Correlations between anthropometric measurements, adipokines and hormonal markers

In both groups, anthropometric measurements (BMI and WHR) correlated positively with plasma leptin levels. However, only abdominal obesity (WHR) showed a significant inverse correlation with adiponectin (table 3). No significant correlations were observed between anthropometric measurements and CSF adipokines or serum vitamin D levels.

**Table 3:** Baseline Spearman’s rank correlation coefficients (ρ) between anthropometric measurements and plasma adipokines

	Leptin	Adiponectin	Lep/Adpn	Resistin	Adipsin
Participants with MS					
BMI	0.84*	−0.22	0.81*	0.37	0.49
WHR	**0.53†**	−0.64*	0.71*	0.29	0.28
Healthy controls					
BMI	**0.53†**	0.01	0.38	0.43	0.24
WHR	0.70*	**−0.62†**	0.79*	0.19	0.11

* Correlation is significant at the 0.01 level (two-tailed).

† Correlation is significant at the 0.05 level (two-tailed).

BMI, body mass index; Lep/Adpn, leptin-to-adiponectin ratio; MS, multiple sclerosis; WHR, waist-to-hip ratio.

Among PwMS, adipokine levels did not correlate with age, MS disease duration, or serum levels of vitamin D, oestradiol, FSH or LH (online supplemental table 2). In contrast, among HCs, serum FSH (ρ = −0.62, p=0.01) and LH (ρ = −0.74, p=0.001) were inversely correlated with resistin levels.

### Correlations of anthropometric measurements, adipokines and vitamin D with inflammatory markers

We next examined the correlations of anthropometric measurements, plasma adipokines and serum vitamin D levels with circulating inflammatory markers (table 4). Among PwMS, both BMI and the leptin-to-adiponectin ratio showed positive correlations with key proinflammatory markers, including hs-CRP, TNF-α and IL-6. In HCs, BMI correlated only with the anti-inflammatory cytokine IL-10, while the leptin-to-adiponectin ratio was positively associated with hs-CRP, IL-1β and IL-6. Notably, inverse correlations of serum vitamin D levels with hs-CRP, TNF-α and IFN-γ levels were observed exclusively in PwMS.

**Table 4:** Baseline Spearman’s rank correlation coefficients (ρ) of anthropometric measurements, plasma adipokines and serum vitamin D with circulating inflammatory biomarkers

	hs-CRP	TNF-α	IFN-γ	CCL2	CXCL8	IL-1β	IL-6	IL-10
Participants with MS								
BMI	0.57*	0.58*	0.49	−0.14	0.25	−0.44	**0.63†**	0.34
WHR	0.50	0.53*	0.43	−0.13	0.42	−0.19	0.50	0.51
Leptin	0.55*	0.52*	0.46	−0.08	0.14	−0.22	0.49	0.34
Adiponectin	−0.19	−0.57*	−0.42	0.14	−0.23	0.11	−0.26	−0.24
Lep/Adpn	0.55*	**0.66†**	0.51*	−0.06	0.31	−0.18	0.51*	0.39
Resistin	0.20	−0.09	0.11	−0.49	−0.06	−0.29	0.34	0.46
Adipsin	**0.64†**	0.37	0.40	0.18	−0.01	0.03	0.26	0.29
Vitamin D	**−0.65†**	**−0.64†**	**−0.64†**	0.33	−0.21	0.28	−0.41	0.20
Healthy controls								
BMI	0.22	0.19	−0.09	0.32	0.16	0.15	0.27	0.54*
WHR	0.49	0.06	−0.21	0.19	−0.25	**0.71†**	0.48	−0.01
Leptin	0.38	0.20	−0.03	0.47	−0.03	0.64*	0.43	0.43
Adiponectin	−0.60*	0.08	0.16	0.03	0.19	−0.37	**−0.76†**	0.09
Lep/Adpn	0.56*	0.03	−0.21	0.34	−0.11	0.64*	**0.78†**	0.16
Resistin	0.50	0.30	0.55*	−0.03	−0.10	−0.05	0.47	0.46
Adipsin	0.33	0.37	0.35	0.00	0.02	0.14	0.31	0.35
Vitamin D	0.05	−0.07	0.11	−0.15	−0.17	−0.07	0.02	−0.38

* Correlation is significant at the 0.05 level (two-tailed).

† Correlation is significant at the 0.01 level (two-tailed).

BMI, body mass index; CCL2, C-C Motif Ligand 2; CXCL8, C-X-C motif chemokine ligand 8; hs-CRP, high-sensitivity C reactive protein; IFN-γ, interferon-γ; IL, interleukin; Lep/Adpn, leptin-to-adiponectin ratio; MS, multiple sclerosis; TNF-α, tumour necrosis factor-α; WHR, waist-to-hip ratio.

The plasma and CSF levels of adipokines were significantly correlated for adiponectin (r=0.87, p=0.002) and adipsin (r=0.90, p<0.001), but not for leptin (r=0.34, p=0.37) or the leptin-to-adiponectin ratio (r=0.19, p=0.62). Resistin was undetectable in the CSF. Correlations between adipokines and inflammatory markers in CSF were limited (online supplemental table 3). Plasma (r=0.77, p=0.02) and CSF (r=0.71, p=0.03) levels of adipsin correlated with CSF CCL2, and plasma resistin level correlated inversely with CSF IFN-γ (r= −0.68, p=0.04). Serum vitamin D levels were inversely correlated with CSF hs-CRP levels (r=−0.80, p=0.01).

### Multivariable associations between BMI, adipokine imbalance and inflammation

Multiple linear regression models were used to assess whether BMI and the leptin-to-adiponectin ratio were independently associated with circulating inflammatory markers, adjusting for age, disease duration, FSH or vitamin D levels as a covariate.

In PwMS, BMI was independently associated with hs-CRP (p<0.001 with age as a covariate; p<0001 with disease duration; p=0.003 with FSH; p=0.02 with vitamin D) and TNF-α (p<0.001, p=0.003, p=0.002, and p=0.03, respectively). The association between BMI and IL-6 remained significant after adjusting for age (p=0.038) and disease duration (p=0.049), but not after adjusting for FSH (p=0.055) or vitamin D (p=0.14). The leptin-to-adiponectin ratio was independently associated with hs-CRP (p=0.007 with age; p=0.007 with disease duration; p=0.01 with FSH and vitamin D) and TNF-α (p=0.001, p=0.003, p=0.002, and p=0.003, respectively), while the associations with IFN-γ and IL-6 were not significant after adjustments.

In HCs, the association between BMI and IL-10 was no longer significant after adjusting for any covariate. Leptin-to-adiponectin ratio remained independently associated with hs-CRP (p<0.001 with age and FSH; p=0.001 with vitamin D) and IL-6 (p<0.001 for all covariates), while its association with IL-1β was lost after adjustment.

### Correlations of anthropometric measurements, adipokines and vitamin D with MS severity

We assessed correlations between anthropometric measurements (BMI and WHR), plasma and CSF adipokine levels, serum vitamin D levels and MS severity indicators, including clinical scores (EDSS, MSSS), brain MRI metrics (LV, WBV) and fluid biomarkers (NfL, GFAP and CHI3L1; table 5, online supplemental table 4 for CSF values).

**Table 5:** Baseline Spearman’s rank correlation coefficients (ρ) of anthropometric measurements, adipokines and serum vitamin D with MS severity biomarkers

	EDSS	MSSS	MRI LV	MRI WBV	s-NfL	s-GFAP	p-CHI3L1
Participants with MS							
BMI	−0.005	0.48	−0.20	0.21	0.06	−0.36	0.21
WHR	0.04	0.36	0.14	−0.03	0.08	−0.03	0.26
p-Leptin	0.08	0.42	0.01	0.06	0.24	−0.19	0.34
p-Adiponectin	0.09	−0.09	0.10	0.04	0.12	−0.11	−0.32
p-Lep/Adpn	0.06	0.46	−0.03	−0.01	0.09	−0.15	0.38
p-Resistin	−0.35	−0.07	−0.13	0.55	−0.15	−0.18	0.16
p-Adipsin	0.26	0.08	0.50	−0.27	0.72*	0.27	**0.54†**
p-Adipsin/BMI	0.42	−0.23	**0.64†**	−0.26	0.79*	**0.52†**	0.44
c-Adipsin	0.43	0.29	**0.78†**	0.02	0.56	0.09	0.05
Vitamin D	0.31	−0.04	0.54	−0.21	0.17	0.07	−0.16

* Correlation is significant at the 0.01 level (two-tailed).

† Correlation is significant at the 0.05 level (two-tailed).

BMI, body mass index; c, cerebrospinal fluid; CHI3L1, chitinase-3-like protein 1; EDSS, Expanded Disability Status Scale; GFAP, glial fibrillar acidic protein; Lep/Adpn, leptin-to-adiponectin ratio; LV, white matter lesion volume; MS, multiple sclerosis; MSSS, Multiple Sclerosis Severity Score; NfL, neurofilament light chain; p, plasma; s, serum; WBV, whole brain volume; WHR, waist-to-hip ratio.

BMI (ρ=0.48, p=0.06) and the leptin-to-adiponectin ratio (ρ=0.46, p=0.07) showed a trend towards a positive correlation with MSSS. Among adipokines, plasma adipsin level was positively correlated with circulating NfL and CHI3L1. BMI-normalised plasma adipsin positively correlated with LV, serum NfL and GFAP, and showed a near-significant correlation with CSF NfL (ρ=0.67, p=0.050). CSF adipsin levels also correlated positively with LV. No significant correlations were observed between other adipokines (absolute or BMI-normalised), vitamin D levels and MS severity measures.

### Longitudinal changes in adipokine levels during MHT

Fourteen PwMS (88%) and 13 HCs (87%) completed the 1-year follow-up with MHT (figure 1). The higher oestradiol dose was used by 79% of PwMS and 62% of HCs for the majority of the follow-up period. As previously reported, MS disease activity remained largely stable, as assessed by relapse rate, EDSS and brain MRI findings.^24^

BMI and WHR data were missing for two PwMS and six HCs at the 12-month follow-up. Among participants with complete data, mean BMI remained stable in PwMS (26.6 at baseline vs 26.4 at 12 months; p=0.77), while a slight, non-significant increase was observed in HCs (26.8 vs 27.5; p=0.09). Mean WHR remained unchanged in both groups over the study period.

**Figure 2:**
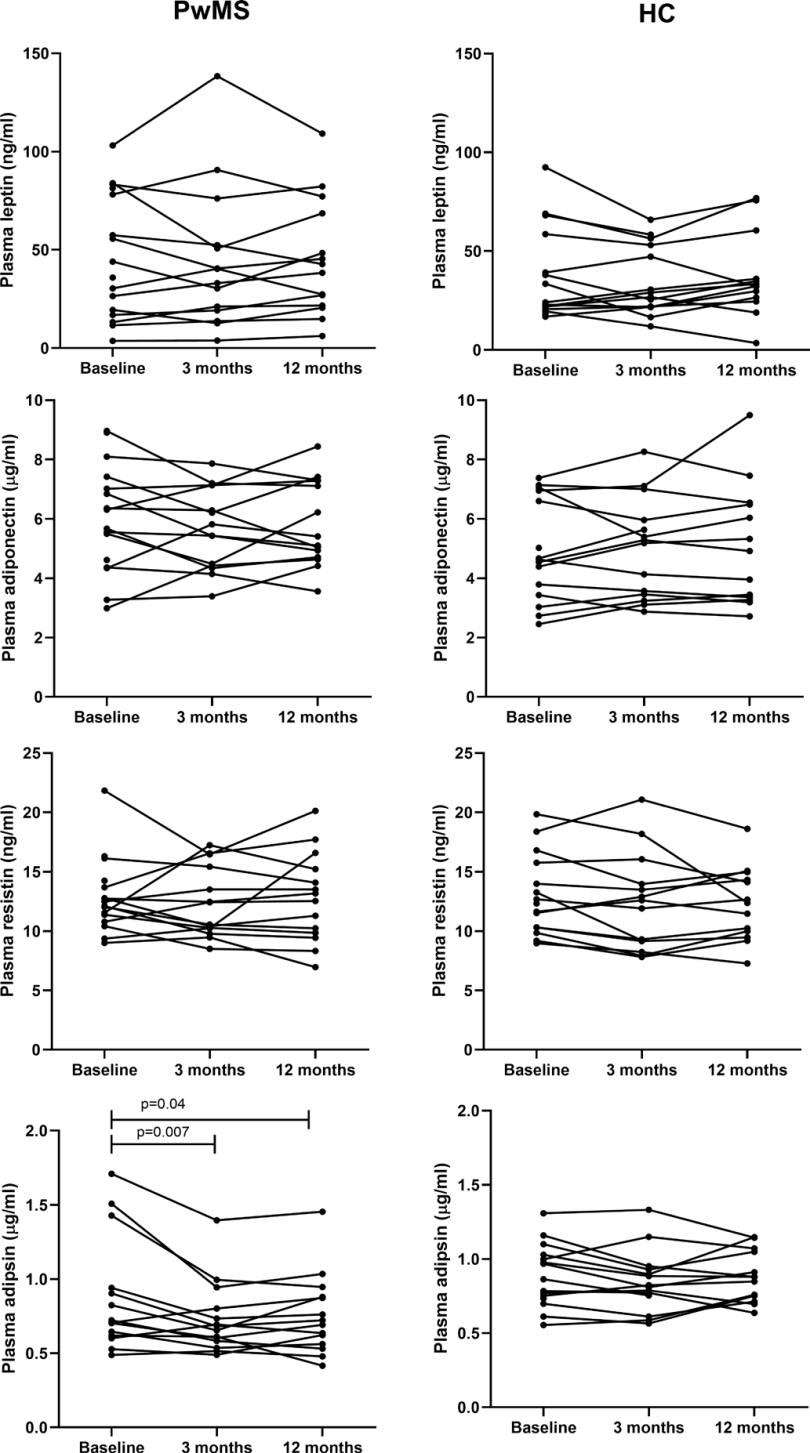
Individual changes in plasma adipokine levels during 1 year of menopausal hormone therapy in participants with multiple sclerosis (PwMS) and healthy controls (HC).

In PwMS, plasma adipsin levels significantly decreased at 3 months (p=0.007), particularly among individuals with higher baseline concentrations (figure 2). This reduction persisted at 12 months (p=0.04). Other plasma adipokines or the leptin-to-adiponectin ratio showed no significant longitudinal changes (figure 2).

Due to the limited number of follow-up CSF samples (n=6), statistical analysis of changes in CSF adipokine levels was not feasible. However, descriptive trends were noted: CSF leptin levels decreased in five PwMS and increased in one participant with a markedly elevated baseline level. CSF adiponectin and adipsin levels decreased in four participants and remained stable in two (online supplemental figure 2).

## Discussion

In our study of menopausal women with MS, higher BMI and an elevated leptin-to-adiponectin ratio were associated with increased levels of inflammatory markers, suggesting a pro-inflammatory metabolic profile. This profile also correlated with a trend towards accelerated disability progression, as reflected by higher MSSS. Interestingly, adipsin—a complement-related adipokine—showed associations with neuroinflammatory biomarkers and was found to decline following MHT, indicating a potential modulatory effect of ovarian hormones on complement system activity.

### Metabolic–immune interactions in MS

Our findings support the hypothesis and previous in vivo observations that link metabolic dysregulation, including obesity and elevated leptin levels, to increased proinflammatory cytokine levels in MS.^25^,^27^ We observed significant positive correlations between elevated BMI, an increased leptin-to-adiponectin ratio, and systemic inflammatory markers, including hs-CRP, TNF-α and IL-6. These associations were independent of age, disease duration, FSH and vitamin D levels, suggesting that adipose tissue may exert a direct proinflammatory effect in menopausal women with MS.

Although baseline adipokine levels did not differ significantly between PwMS and HCs, their associations with key pro-inflammatory markers—particularly TNF-α—were more pronounced in the MS group. This suggests a disease-specific inflammatory response that may reflect heightened immune sensitivity to metabolic cues in MS. Furthermore, MS-related inflammation may reciprocally stimulate adipose tissue to release additional proinflammatory adipokines, potentially establishing a self-perpetuating inflammatory loop.^28^ This metabolic-inflammatory axis may contribute to disease progression by sustaining chronic peripheral immune activation which further exacerbates neuroinflammation and neurodegeneration and could be highly relevant during menopausal transition.^9 10^

### Adipsin as a link between metabolism, inflammation and MS severity

Adipsin, also known as complement factor D, is primarily secreted by adipocytes but is also produced by monocytes and macrophages. It plays a central role in activating the alternative complement pathway, leading to the generation of complement component C3. Adipsin has been implicated in promoting inflammation and endothelial dysfunction, with elevated levels associated with increased cardiovascular risk and all-cause mortality.^29^

While we observed a trend linking higher BMI and leptin-to-adiponectin ratio to more rapid disability progression, the most robust associations with MS severity markers were observed for adipsin. Plasma adipsin levels showed strong positive correlations with cerebral white matter lesion load and several circulating biomarkers indicative of MS activity and progression.^21^ All associations between adipsin and disease activity markers—except for CHI3L1—were strengthened after BMI-normalisation of adipsin values. This aligns with our previous findings in RRMS, where elevated BMI-normalised plasma adipsin was associated with greater disease activity, disability and lesion burden,^16^ implying that adipsin may have independent regulatory roles beyond just reflecting age or adiposity.^30^ The role of adipsin in MS remains underexplored, but as such, adipsin may represent a promising biomarker for metabolic–immune crosstalk, particularly in hormonally dynamic female populations.

Observed reduction in plasma adipsin levels during oral MHT may suggest a potential modulatory effect of oestrogen on complement system activity, linking hormonal modulation to immune-metabolic rebalancing. Future studies should include simultaneous measurement of adipsin, C3 and other complement components to clarify whether reduced adipsin levels during MHT reflect decreased activation or increased consumption—potentially revealing novel immunometabolic mechanisms and therapeutic opportunities in modulating disease activity and progression.

### Vitamin D and inflammation

Vitamin D has well-established anti-inflammatory properties, and low levels have been associated with increased risk and activity of MS.^31^ In our cohort, serum 25(OH)D concentrations were predominantly sufficient and significantly higher in PwMS than in HC, likely reflecting more intensive supplement use. Notably, strong inverse correlations between circulating vitamin D and key proinflammatory markers were observed exclusively in PwMS, suggesting a closer link between vitamin D status and systemic inflammation in the context of MS-related immune dysregulation. While the potential influence of supplementation bias must be acknowledged, these findings support the value of maintaining adequate vitamin D levels as part of holistic care in women with MS.

Oestrogen has been demonstrated to enhance vitamin D function by promoting its accumulation and increasing the expression of vitamin D receptor.^32^ Stronger immunomodulatory effects of vitamin D have been demonstrated in women with MS compared with men, and preclinical studies indicate that vitamin D suppresses neuroinflammation only in the presence of oestrogen.^33^,^35^ While human data remain limited, this raises the possibility that declining oestrogen levels during menopause may attenuate the anti-inflammatory potential of vitamin D.^32^ No major clinical trials have directly studied the interaction between vitamin D and MHT in MS; this could represent a promising therapeutic target for future investigation.

### Integrating hormonal and metabolic context in MS biomarker profiling

MS involves immune dysregulation, neuroinflammation, neurodegeneration and environmental factors, such as vitamin D deficiency.^10^ Given its multifactorial nature, biomarker strategies should reflect age-specific contexts such as menopause-related obesity, where inflammatory and metabolic pathways intersect. Incorporating variables like age, body weight and hormonal status is essential for accurate biomarker interpretation.

Menopause alters the hormonal milieu, affecting biomarker dynamics and necessitating personalised approaches. Immunomodulatory therapies further complicate interpretation, underscoring the need for integrated profiling. Combining CSF and blood biomarkers with neuroimaging may enhance precision in MS management.^36^

### Strengths, limitations and future perspectives

A key strength of this study lies in the homogeneity of the study population with respect to menopausal phase, age range and the use of a consistent MHT regimen. This uniformity is particularly valuable when examining immunometabolic markers such as adipsin, which has been shown to increase with age and may be influenced by immunosenescence.^37^ By minimising variability in hormonal and demographic factors, the study design enhances the interpretability of the observed associations between metabolic markers and inflammatory activity.

However, several limitations must be acknowledged. A greater proportion of PwMS than HC were classified as postmenopausal; although this difference did not reach statistical significance, a potential confounding effect on baseline comparisons cannot be excluded. In MS, the generalisability of these findings is limited to perimenopausal and early postmenopausal women with low inflammatory activity and mild to moderate disability. The relatively small sample size may have limited the statistical power to detect certain associations—particularly in CSF analyses—and may have increased the risk of overestimating others. Additionally, multiple comparisons increase the possibility of false-positive findings. To mitigate this, we focused on identifying consistent patterns across multiple biomarkers rather than relying solely on isolated statistically significant results. Correlations lacking support from related measures were interpreted with caution, even when statistically significant.

Multivariate models and subgroup analyses based on MS subtype, DMT use or oestradiol dose were not feasible in this study. The nature of inflammation evolves over the MS disease course and becomes partly compartmentalised in the CNS with disease progression.^38^ Therefore, relapsing and progressive subtypes should ideally be considered separately, although the transition from RRMS to SPMS is a continuum and rigorous distinction between these subtypes can be difficult. Furthermore, DMTs can affect both immune and metabolic pathways, including adipokine and inflammatory cytokine signalling; their potential confounding effects cannot be excluded. Although data on specific DMT effects on adipokines remain limited, treatments such as interferon-β and dimethyl fumarate may alter adipose tissue metabolism.^14^ However, exclusion of all DMT-treated patients would have made recruitment much more difficult and would not have properly represented this population, as virtually all patients with active MS are on DMTs. Future studies should involve stratification by DMT class and treatment status to better isolate biomarker effects and account for off-target influences on immune and metabolic pathways.^39^ Additionally, menopausal staging should be done precisely according to standardised definitions, and detailed information on vitamin D supplementation and interindividual variation in endogenous regulation should be collected and incorporated into analyses.

## Conclusions

This study highlights the interplay between obesity, adipokine dysregulation and systemic inflammation in menopausal women with MS. Elevated BMI and leptin-to-adiponectin ratios were linked to increased inflammatory markers and a trend towards faster disability progression. Among the adipokines, adipsin emerged as a promising biomarker, showing strong associations with lesion burden and fluid markers, independent of BMI. Its reduction during MHT suggests a potential immunomodulatory role involving the complement system. While limited by sample size and lack of subgroup analyses, these findings support further investigation into immunometabolic mechanisms and the therapeutic potential of targeting adipokines and complement pathways in MS.

## Supplementary material

10.1136/bmjno-2025-001295 online supplemental file 1

## Data Availability

Data are available on reasonable request.
