# Supplementary material for: Immunometabolic profiling in menopausal women with multiple sclerosis: the role of adipokines and hormone therapy
Source: BMJ Neurol Open. 2025 Nov 30;7(2):e001295. doi: 10.1136/bmjno-2025-001295 (PMC12684142; doi:10.1136/bmjno-2025-001295)
Supplement: online supplemental file 1 [file bmjno-7-2-s001.pdf]

Supplementary Table 1. BMI-normalised plasma adipokine levels at baseline presented as median (interquartile range).

	Participants with MS (*n* = 16)	Healthy controls (*n* = 15)	p-value[1]
**Leptin/BMI**	1.50 (0.76–2.41)	1.02 (0.81–2.06)	0.57
**Adiponectin/BMI**	0.21 (0.15–0.30)	0.17 (0.13–0.26)	0.14
**Resistin/BMI**	0.46 (0.39–0.50)	0.49 (0.40–0.53)	0.52
**Adipsin/BMI**	0.027 (0.023–0.035)	0.032 (0.028–0.039)	0.32

MS, multiple sclerosis

[1] Mann-Whitney U test. Level of significance $p < 0.05$.

Supplementary Table 2. Baseline Spearman's rank correlation coefficients (rho) of plasma and CSF adipokines, age, MS disease duration, serum vitamin D and hormones. Resistin was undetectable in the CSF.

	Age	Disease duration	Vitamin D	E2	FSH	LH
		**Participants with MS**				
**Plasma leptin**	−0.12	−0.29	−0.11	0.08	0.11	0.23
**CSF leptin**	−0.25	−0.25	0.05	0.54	−0.34	−0.17
**Plasma adiponectin**	−0.11	−0.03	0.27	0.04	0.08	−0.02
**CSF adiponectin**	0.08	0.15	0.13	0.20	−0.08	0.13
**Plasma lep/adpn**	−0.14	−0.29	−0.17	0.07	0.03	0.11
**CSF lep/adpn**	−0.00	−0.09	0.29	0.17	−0.08	0.03
**Plasma resistin**	−0.01	−0.22	0.21	0.29	−0.27	−0.04
**Plasma adipsin**	0.35	0.25	−0.03	−0.21	0.25	0.45
**CSF adipsin**	0.06	0.12	0.12	0.28	−0.03	0.25
		**Healthy controls**				
**Plasma leptin**	−0.09	.	0.12	0.04	−0.13	−0.22
**Plasma adiponectin**	0.16	.	−0.15	0.08	0.13	0.13
**Plasma lep/adpn**	−0.19	.	0.03	−0.12	−0.04	−0.13
**Plasma resistin**	−0.18	.	0.16	0.27	**−0.62***	**−0.74****
**Plasma adipsin**	−0.04	.	0.06	0.18	−0.32	−0.36

CSF, cerebrospinal fluid; MS, multiple sclerosis: E2, estradiol; FSH, follicle-stimulating hormone; LH, luteinising hormone; leptin-to-adiponectin ratio

** Correlation is significant at the 0.01 level (2-tailed).

* Correlation is significant at the 0.05 level (2-tailed).

Supplementary Table 3. Baseline Spearman's rank correlation coefficients (rho) of plasma/CSF adipokines and serum vitamin D with CSF levels of inflammatory biomarkers in participants with MS (n = 9). Resistin was undetectable in the CSF.

	Participants with MS							
	CSF hs-CRP	CSF TNF-α	CSF IFN-γ	CSF CCL2	CSF CXCL8	CSF IL-1β	CSF IL-6	CSF IL-10
**Plasma leptin**	0.43	−0.18	−0.35	0.38	−0.23	0.13	0.53	0.05
**CSF leptin**	0.12	0.15	0.22	0.42	−0.08	−0.15	−0.07	0.25
**Plasma adiponectin**	−0.13	0.12	−0.45	0.12	0.03	−0.12	0.07	0.08
**CSF adiponectin**	0.27	0.43	−0.50	0.36	0.27	−0.15	0.21	0.38
**Plasma lep/adpn**	0.47	−0.20	−0.13	0.40	−0.27	0.00	0.32	0.02
**CSF lep/adpn**	−0.36	−0.12	0.42	−0.08	−0.03	0.12	−0.22	0.08
**Plasma resistin**	0.10	−0.50	−0.68*	0.03	−0.18	0.10	0.62	−0.30
**Plasma adipsin**	0.43	0.50	−0.15	**0.77***	0.28	0.10	0.37	0.35
**CSF adipsin**	0.44	0.44	−0.44	**0.71***	0.31	0.07	0.54	0.37
**Serum vitamin D**	**−0.80****	−0.40	−0.22	−0.12	−0.10	0.25	−0.05	−0.38

CSF, cerebrospinal fluid; MS, multiple sclerosis; hs-CRP, high-sensitivity C-reactive protein; TNF-α, tumor necrosis factor-α; IFN-γ, interferon-γ; CCL2, C-C Motif Ligand 2; CXCL8, C-X-C motif chemokine ligand 8; IL, interleukin; lep/adpn, leptin-to-adiponectin ratio

** Correlation is significant at the 0.01 level (2-tailed).

* Correlation is significant at the 0.05 level (2-tailed).

Supplementary Table 4. Baseline Spearman's rank correlation coefficients (rho) of anthropometric measurements, adipokines, and vitamin D with MS severity biomarkers in CSF.

	Participants with MS		
	CSF NfL	CSF GFAP	CSF CHI3L1
**BMI**	–0.35	–0.50	–0.58
**WHR**	–0.02	0.12	0.05
**Plasma leptin**	–0.15	–0.33	–0.20
**CSF leptin**	0.29	–0.25	0.05
**Plasma adiponectin**	0.27	0.10	0.10
**CSF adiponectin**	0.40	0.36	0.08
**Plasma lep/adpn**	–0.25	–0.35	–0.33
**CSF lep/adpn**	0.09	–0.42	0.18
**Plasma resistin**	–0.63	–0.18	–0.30
**Plasma adipsin**	0.48	0.43	–0.03
**Plasma adipsin/BMI**	0.67	0.52	0.30
**CSF adipsin**	0.39	0.41	0.00
**Serum vitamin D**	–0.07	–0.17	–0.02

MS, multiple sclerosis; CSF, cerebrospinal fluid; BMI, body mass index; WHR, waist-hip ratio; NfL, neurofilament light chain; GFAP, glial fibrillary acidic protein; CHI3L1, chitinase-3-like protein 1; Lep/Adpn, leptin-to-adiponectin ratio.

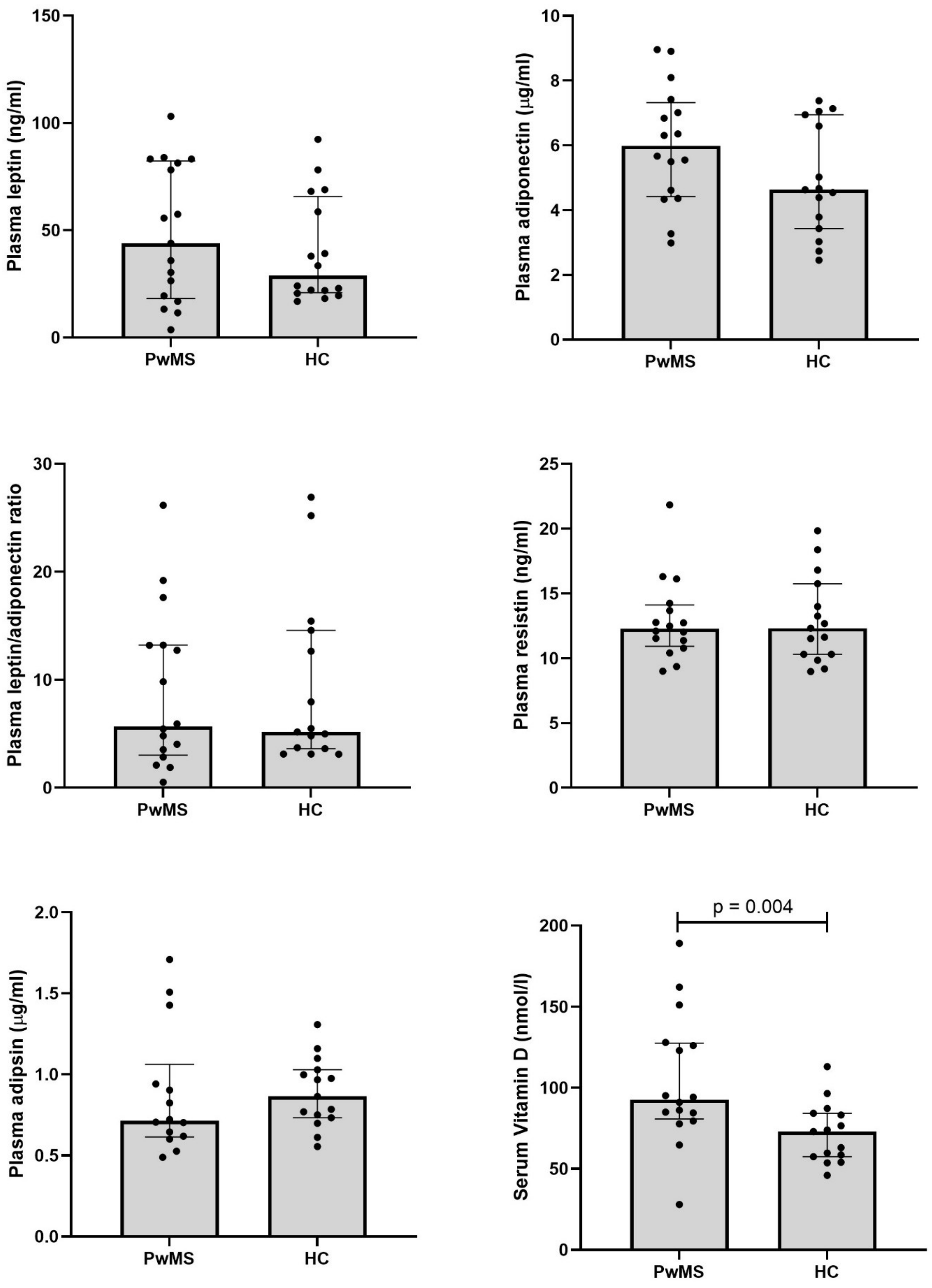

Supplementary Figure 1. Plasma adipokines and serum vitamin D levels at baseline in participants with MS (PwMS) and healthy controls (HC).

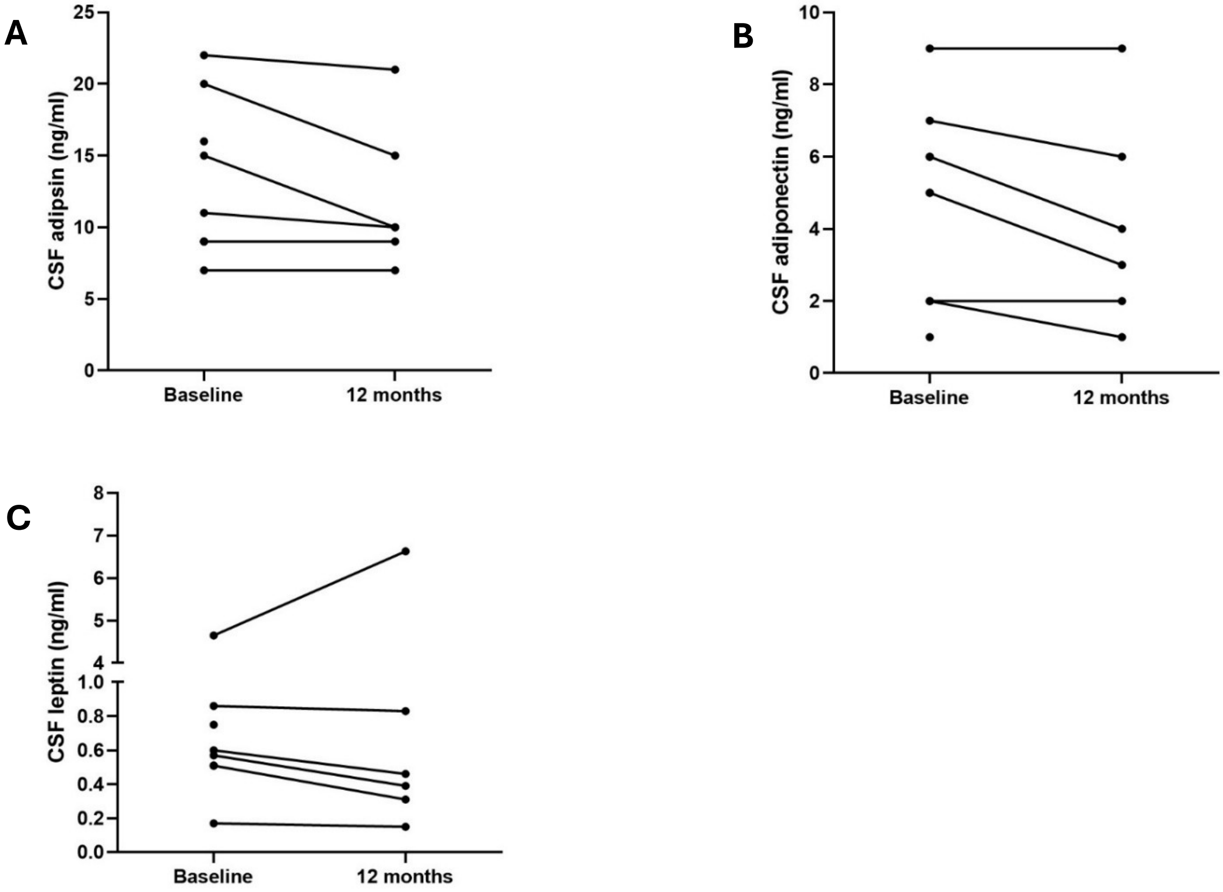

Supplementary Figure 2. Individual changes in cerebrospinal fluid (CSF) levels of A) adipsin, B) adiponectin and C) leptin during one year of menopausal hormone therapy in participants with MS.
